# Effectiveness of a pain science education programme in middle school students: a randomised controlled trial

**DOI:** 10.3389/fpubh.2024.1423716

**Published:** 2025-01-22

**Authors:** Laura Menés Fernández, Isabel Salvat, Cristina Adillón

**Affiliations:** Department of Medicine and Surgery, Institut Investigation Sanitarian Pere Virgili, Universitat Rovira i Virgili, Reus, Spain

**Keywords:** Pain, pain education, school, children, health literacy

## Abstract

**Introduction:**

Chronic pain in childhood is a prevalent issue affecting approximately 20% of young people aged 8–16 years. This condition negatively impacts their quality of life, as well as their physical, psychological, and social functioning. In recent years, Pain Science Education has emerged as a promising strategy to enhance the conceptualization of pain and mitigate maladaptive pain-related behaviours in children and teenagers. The primary aim of this study is to assess the effectiveness of the Pain Science Education programme called “Learning Pain” in improving the conceptualization of pain amongst 11–13-year-old children enrolled in their first year of middle school.

**Materials and methods:**

A randomised controlled trial with single-blind parallel groups was conducted. Five participating institutes were randomly assigned to either the experimental group, which received the Pain Science Education intervention through the “Learning Pain” programme consisting of two sessions spaced 1 month apart, or the control group, which underwent only study assessments. Both groups underwent initial assessment and follow-up evaluations at 1 month (short term), 7 months (medium-term), and 13 months (long-term). The main outcome measure was the conceptualization of pain, assessed using the Conceptualization of Pain Questionnaire (COPAQ).

**Results:**

The “Learning Pain” programme, a specific Pain Science Education intervention, demonstrated effectiveness in improving the conceptualization of pain at 1, 7, and 13 months follow-up assessments.

**Discussion:**

The “Learning Pain” programme, a specific Pain Science Education intervention, enhances the conceptualization of pain in adolescents aged 11–13 years over short, medium, and long-term periods.

## 1 Introduction

Chronic or persistent pain is defined as pain that lasts more than 3 months (1). Nowadays, it is a common problem in childhood and adolescence with an estimated prevalence of 20% (2–4). This type of pain is the first reason for the high demand for medical care in childhood (5–7) and impacts several areas of children’s lives (8), affecting social life (9), academic performance, and generating school absenteeism (2). It also has negative consequences on the child’s self-esteem and social functioning (10, 11).

The treatment of chronic pain is complex, and although it is currently approaching in an interdisciplinary manner, changing the way people understand pain is an essential part of the treatment (12, 13) as understanding the pain decreases its threat value and favours the choice of more adaptive and effective pain coping strategies (14). Unhelpful pain beliefs or misconceptions about pain are crucial in the progression from acute to chronic musculoskeletal pain. Studies show these beliefs predict the onset of disabling pain in pain-free individuals and the severity of disability over time in those with acute pain (15).

For this reason, Pain Science Education, which has the main objective of this approach to change misconceptions and misbeliefs about pain and its function through an explanatory model (13, 16), emerges as an essential aspect of the approach to chronic pain. This type of intervention is effective in chronic pain conditions in adults (17–20) and in children (21–25). Given that a poor conceptualization of pain plays a significant role in the transition from acute to chronic pain (26, 27), it is essential to promote the contemporary pain paradigm aimed at challenging and correcting these misconceptions through Public health campaigns (28, 29).

Despite these advances, myths still exist around some chronic pain conditions (30–32), and pain knowledge remains outdated compared to contemporary understanding of pain in both children and adults (33, 34). In children and adolescents, when asked why they think they have pain, a high percentage of explanations about the cause of pain are observed that are not related to tissue damage events. This tendency intensifies with age and as pain persists (35). Although this might suggest a more biopsychosocial understanding of pain in children, it is important to question the increasing normalisation of perceiving pain in situations that do not represent a danger to the body. These beliefs may create a significant barrier to good practise in the management of chronic pain (15).

From the age of 11 years, young people begin to have the capacity for abstract thinking and understanding of complex concepts (36). Given the cognitive and affective development at this stage of life, it is a key moment in shaping the beliefs of future adults (37). Therefore, educational interventions targeting pain beliefs could have broad benefits and be an easy, inexpensive, and novel strategy for addressing chronic pain. Public misconceptions grounded in the biomedical model might significantly contribute to the ongoing rise of non-evidence-based pain treatments (38).

Public health campaigns, especially in school settings aimed at young people, emerge as an effective strategy for improving knowledge, beliefs, and attitudes aligned with the contemporary pain paradigm (34, 39, 40). Although there is no research available yet that indicates which type of methodology is most effective for these group interventions in the school setting, those that have used interactive methodologies are better at transferring learning to students’ experiences compared to those using only expository methodologies (24).

However, the number of school-based studies investigating the impact of Pain Science Education on school children is small and more controlled trials with longer-term follow-up are needed to investigate the effectiveness of this intervention before any firm recommendations can be made (40). Moreover, there is only one study on Pain Science Education in Spain (41), which highlights the need for further exploration in this field.

This study will be the first school-based study in Spain examining the impacts of Pain Science Education on pain conceptualization in the long term. The study aimed (a) to analyse whether children had a more accurate conceptualization of pain following the Pain Science Education programme in school children aged 11–13 years and (b) to compare the pain conceptualization accuracy of children who received the intervention versus the control condition. It was hypothesised that the specific Pain Science Education programme for students aged 11–13 years improves the conceptualization of pain in the short (1 month), medium (7 months), and long term (13 months).

## 2 Materials and methods

### 2.1 Study design

This is a single-blind, randomised clinical trial conducted from February 2022 to April 2023 (14 months) following the CONSORT (Consolidated Standards of Reporting Trials) guidelines (42). Due to the nature of the study, it was not feasible to blind the participants; however, the assessor was blinded. The protocol for this study was approved by the Comitè ètic d’Investigació en Persones, Societat i Medi Ambient de la Universitat Rovira i Virgili (CEIPSA) (reference number CEIPSA-2021-TD-0017) and was registered in ClinicalTrials.gov (NCT06060431).

As the participants were minors, informed consent was obtained from their legal guardians, and verbal permission was obtained from the participants before completing the questionnaire.

### 2.2 Participants

The participants in this study were boys and girls between 11 and 13 years old enrolled in the first year of Obligatory Secondary Education in five schools in the municipality of Vilanova i la Geltrú (Barcelona). The inclusion criteria for the study were the following: (a) be able to read, write, and speak Catalan; (b) to be between 11 and 13 years old, inclusive; (c) be in the first year of Obligatory Secondary Education; and (d) have the informed consent and information sheet signed by the legal guardians. The exclusion criteria for the study were as follows: (a) having an intellectual disability or cognitive impairment that interferes with their participation; and (b) not wishing to participate voluntarily in the study. Those participants who did not meet the inclusion criteria or presented any exclusion criteria were excluded from participation in the study. This was done with the collaboration of the tutors of the schools.

In addition, the following elimination criteria were considered during the study: (a) failure to attend 50% of the intervention sessions; (b) failure to attend the day of the assessments; and (c) voluntary withdrawal during the study.

A pilot study was conducted prior to the clinical trial. Based on the results of the pilot, a sample size calculation was performed. So, 88 participants (44 per group) were required to detect a difference of 1.79 points in pain conceptualisation using the Conceptualization of Pain Questionnaire, a standard deviation of 2.56 and given a power of 90% and a significance level of 5%. After increasing the sample size by 20% to cover possible losses during the study, the required sample was 106 participants. These were also distributed in a 1:1 allocation ratio (53 participants in the experimental group and 53 participants in the control group).

### 2.3 Randomisation

The schools participating in the study were assigned a numerical code in order of inclusion. Then, using the EPIDAT programme (Xunta de Galicia), a simple random assignment was made with balanced groups in two groups: the experimental group and the control group.

### 2.4 Outcomes

The main outcome measure was the conceptualization of pain, assessed using the Conceptualization of Pain Questionnaire (COPAQ). This tool is designed to numerically assess pain conceptualization in children and adolescents (43). The questionnaire consists of 15 statements, each with three possible options: “True,” “False” and “Do not know.” Each correct answer counts as one point, and a total score can be obtained ranging from 0 to 15. The questions answered with “do not know” were scored as 0 for the corresponding item. This questionnaire has shown good fit and internal consistency in the age range of 8–17 years old (43).

The independent variables were demographic information, including sex (female/male) and age (years). In addition, participants were asked whether they had experienced pain (yes/no). If participants reported pain, they were classified into acute pain or chronic pain (based on the definition of chronic pain as persistent or recurring pain or pain lasting longer than 3 months) (1, 44).

### 2.5 Data collection

The participants were asked to fill out a questionnaire where personal data (sex and age) and different questions related to pain (presence of current pain or pain in the last month and its persistence) were collected. Pain experience was assessed with the question, “Are you in pain or have you had pain in the last month?.” The possible responses were “Yes” or “No.” Pain duration was assessed with the question, “How long have you had this pain?” which the possible responses being “only a few days,” “less than a 1 month,” “1–3 months,” “3–6 months,” or “more than 6 months.” Finally, participants were asked to fill out the Conceptualization of Pain Questionnaire (COPAQ). The questionnaire was administered to schoolchildren who attended the participating school, whose parents had signed the informed consent, and who assented to participate just before the questionnaire was administered. In the presence of the teachers, the researchers administered both questionnaires. The researchers and the teachers ensured that the students did not discuss their responses with each other.

### 2.6 Learning pain programme

The content was based on the books *Explain Pain* (45) and *Why Do I Hurt?* (46), and examples from the book *Cuentos Analgésicos* (47) were used. For explaining the learning processes of pain, special emphasis was placed on social or modelling learning (48, 49). The most frequent explanations not associated with tissue damage amongst young people were addressed (35). In addition, current evidence on pain education programmes for paediatric ages was considered (21–25, 34). The timing was established considering other similar programmes (12, 16, 34). The language was adapted to the vocabulary and cognitive abilities of the target age group, as suggested by the literature (50).

The programme methodology was designed with the aim of promoting conceptual change, i.e., the “Learning Pain” programme focuses specifically on challenging existing knowledge and knowledge structures, rather than simply learning new information (16). Expository methods (lectures), gamification (role-playing), and guided methods (problem-based learning) were chosen, as they are amongst the most effective and are based on learning neuroscience (51–54). For the guided methodology, a single cooperative activity per subgroup was developed, based on an adaptation of *Protectometer* (55) and the exercise proposals from the book *Cognitive-Behavioral Therapy for Chronic Pain in Children and Adolescents* (56).

The main considerations in planning the activities were practise in retrieval, promoting understanding-based learning, learning transfer, and the importance of activating prior knowledge to learn new concepts (54). Thus, two sessions (Session 1 and Session 2) were designed, with a 4-week interval between them.

### 2.7 Intervention

#### 2.7.1 Experimental group

This group participates in a specific programme in Pain Science Education called “Learning Pain.” Before starting the educational programme, meetings were held with the students’ tutors to explain the contents and objectives of the education programme. The most important concepts to be covered were discussed, and dates for the programme were agreed upon with each school.

The education programme was delivered by the investigators between February and March 2022 in the classrooms of the schools. The programme consisted of two sessions: the first lasting 90 min and the second lasting 60 min, held 1 month later. The programme contents were aligned with the target concepts of Pain Science Education interventions for children and adolescents: “Pain is a protector,” “Pain is not an accurate marker of tissue state,” “Pain is a brain output,” “There are many potential contributors to anyone’s pain” and “We are all bioplastic” and “The pain system can become overprotective” and “Pain education is treatment” (57).

The first session provided information on the concept of pain from the current neuroscience paradigm. Emphasis was placed on the biological function of pain and on differentiating the concepts of tissue damage and pain. The session was structured using learning methodologies that combined a master class and gamification. The master class included a PowerPoint presentation that covered content in line with the learning objectives that “Pain is a protector,” “Pain is not an accurate marker of tissue state,” and “Pain is a brain output” (57). Additionally, other objectives such as “There are many potential contributors to anyone’s pain “and “We are all bioplastic” (38) were addressed through gamification, specifically a role-playing game that was developed for the current project.

In the role-playing game, participants were divided into two equal groups (12–14 people each) representing the brain (scenario) of two different people. Each group was further subdivided (3–5 people) so that participants formed four departments (4 roles) by using the metaphor of the brain functioning as a company, a concept that has been used in other education programmes (12).The departments included: the danger message, the context, the person’s learning and the head department. Each department had different response options that they had to fill in on a Control Panel (Supplementary Appendix 1) to conclude whether they would elicit pain in the person they represented. Three different scenarios were presented: two involving tissue damage and one without tissue damage. After each scenario, the responses of both groups were discussed.

In the second session, the most frequent explanations not associated with tissue damage amongst young people were addressed (25). The main objective of this session was to define harm and reduce the threat value of different situations related to explanations not associated with tissue damage, addressing learning objectives (57) “The pain system can become overprotective “and “Pain education is treatment.” For this purpose, a problem-solving activity was carried out through a dynamic in which participants, in groups, had to classify different situations as to whether they were dangerous or not for the integrity of the organism on specific panel designed for this purpose (Supplementary Appendix 2).

The Conceptualization of Pain Questionnaire (COPAQ) was administered at the beginning of the study (COPAQ-baseline), 1 month after baseline at the 1-month assessment (COPAQ-1 month), and at follow-up at the 7-month assessment (COPAQ-7 months) and the 13-month assessment (COPAQ-13 months) (Supplementary Appendix 3).

#### 2.7.2 Control group

The participants in the control group continued with the established curricular teaching programme which does not include any specific pain-related teaching content. They only underwent the initial assessment, 1 month, 7 months, and 13 months (Supplementary Appendix 3).

### 2.8 Data processing and analysis

Statistical analyses were performed using SPSS version 26.0 (Statistical Package for the Social Sciences for Windows; IBM Corp, Armonk, NY, United States). Normally distributed data for continuous variables were summarised with means and standard deviations (SD). Demographic variables were described as absolute frequencies and percentages. To analyse the conceptualization of pain between groups (experimental and control), the chi-square test and independent-sample Student’s *t*-test were used. For all tests, *p*-values were two-sided. A *p*-value less than 0.05 was considered significant.

To evaluate the effects of the intervention over time and between groups, linear mixed models (LMM) analyses were conducted. This approach allows for the analysis of longitudinal data by considering both between-subject and within-subject variations over time. The model variables included the COPAQ questionnaire score as the dependent variable and the factors Group (experimental vs. control) and Time (0, 1, 7, 13 months). Fixed effects were included for the factors Group and Time, as well as for their interaction. Additionally, the repeated effects of Time were modelled using an unstructured covariance structure.

The analyses were performed using SPSS software version 27. In the SPSS linear mixed models menu, the dependent variable (COPAQ) was specified, and Group and Time were designated as fixed factors. The subject identifier (ID) was used to specify the subject variable. An unstructured covariance structure was selected to model the repeated effects of Time. The Restricted Maximum Likelihood (REML) method was used to estimate the model parameters, and the Satterthwaite method was applied for degrees of freedom correction. The fixed effects of Group, Time, and their interaction were evaluated using Type III fixed effects tests, reporting *F*-values and significance levels (*p*-values) for each effect.

The estimated marginal means for COPAQ scores at each time point were calculated and reported. Additionally, various information criteria (AIC, BIC, CAIC) were used to assess the model fit. The results of the linear mixed models indicated significant effects for both the Group and Time factors, as well as for their interaction. These findings suggest that the intervention had a significant impact on COPAQ questionnaire scores over time in the experimental group compared to the control group. This methodology provides a robust and detailed evaluation of longitudinal and between-group effects, offering a more comprehensive understanding of the intervention’s effectiveness.

## 3 Results

### 3.1 Description of the sample

A total of 422 students enrolled in the first year of middle school in the 2021–2022 academic year (5 schools) were evaluated for selection. Of these, 99 were excluded; 87 of them refused to participate and the remaining 12 were excluded for not meeting the eligibility criteria (9 for presenting intellectual disability and 3 for not giving consent), leaving a total of 323 participants included in the study (Supplementary Appendix 4). Randomisation distributed the schools into two groups, leaving 3 in the experimental group and the remaining 2 in the control group. Thus, the experimental group consisted of 191 participants and the control group of 132.

The mean (standard deviation) age is 12.02 (0.26) years old, 82% currently experienced pain or had pain in the past month, and 46% of the participants are female (Table 1).

**Table 1:** General descriptive characteristics of the participants.

Characteristics		Experimental group (*n* = 163)	Control group (*n* = 130)	*p*-value
Age		12.03 (0.303)	12.05 (0.211)	0.990
Sex	Female	71 (44%)	64 (49%)	0.333
	Male	92 (57%)	66 (51%)	
Pain in past month	Yes	134 (82%)	106 (82%)	0.990
	No	30 (18%)	24 (18%)	
Pain duration	Acute pain	93 (70%)	74 (69%)	0.985
	Chronic pain	41 (30%)	32 (31%)	

Data are expressed as mean (standard deviation), and number (percentage). The *p*-value has been obtained using the T-Student test or Chi-square test. *p <  0.05.

### 3.2 Conceptualization of pain

In the initial assessment of the conceptualization of pain through the correct answers on the COPAQ-baseline questionnaire, the result is similar in both groups, with the average number of correct answers being slightly higher in the experimental group. However, the difference is not statistically significant (*p* = 0.125) (Table 2).

**Table 2:** Mean number of correct answers in the COPAQ questionnaire.

	Experimental group (*n* = 163)	Control group (*n* = 130)	*p*-value
COPAQ- baseline	6.83 (1.80)	6.45 (2.31)	0.125
COPAQ-1 months	8.70 (2.85)	6.91 (2.28)	0.000*
COPAQ-7 months	9.17 (3.05)	6.92 (2.25)	0.000*
COPAQ-13 months	9.02 (2.82)	6.81 (2.03)	0.000*

Data are expressed as mean (standard deviation). The *p*-value has been obtained using the T-Student test. *p-value < 0.05.

As for the results of the 1-month assessment, as shown in Table 2, there is an increase in the number of correct answers to the COPAQ-1-month questions, resulting in a statistically significant difference between groups (*p* < 0.001). These differences were maintained in the 7-month assessment (COPAQ-7 months), with an increase in the mean number of correct answers in the experimental group, compared to the control group (*p* < 0.001).

In the 13-month assessment, the average number of correct answers on the COPAQ-13 months decreases in both groups, although it continues to be higher in the experimental group. This difference is statistically significant (*p* < 0.001).

**Figure 1:**
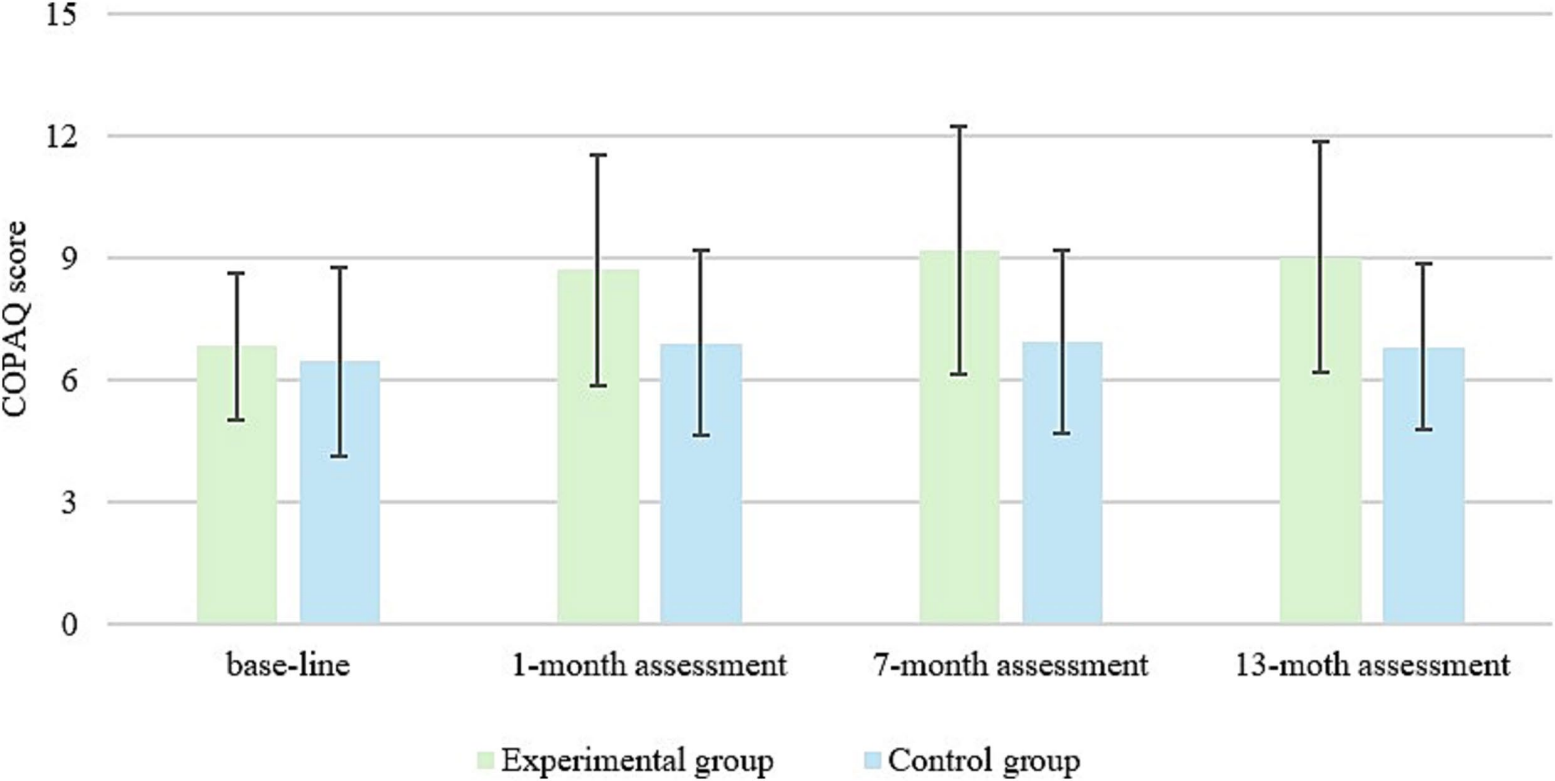
Change in the conceptualization of pain between the experimental and control groups.

The change in the conceptualization of pain between the two groups can be seen in Figure 1.

No statistically significant differences were found between sex in any of the COPAQ questionnaires (T-student; *p* > 0.05).

### 3.3 Mixed linear models analyse

Linear mixed models were used to evaluate COPAQ questionnaire scores over time and between the experimental and control groups. Below, we present the obtained results.

#### 3.3.1 Information criteria

The information criteria used to assess the model fit were as follows:

- Restricted Log Likelihood (−2LL): 4293.711
- Akaike Information Criterion (AIC): 4313.711
- Hurvich and Tsai Criterion (AICC): 4313.902
- Bozdogan Criterion (CAIC): 4374.333
- Schwarz Bayesian Criterion (BIC): 4364.333

These values suggest a good model fit to the data, as they are lower than the comparative values of other possible models (if available).

#### 3.3.2 Type III fixed effects

Fixed effects were evaluated for the factors of group and time, as well as their interaction. The results are shown in Table 3.

**Table 3:** Analysis type III fixed effects.

Source	Num df	Residual df	F	Sig.
Intercept	1	279.980	3464.520	<0.001
Group	1	291.000	16.508	<0.001
Time	3	290.000	31.040	<0.001

These results indicate that both the group and time have a significant effect on COPAQ questionnaire scores.

#### 3.3.3 Estimated marginal means

The estimated marginal means for COPAQ scores at different time points are shown in Table 4.

**Table 4:** Analysis of estimated marginal means, standard errors, and confidence intervals across time intervals assessments.

Time	Mean	Standard error	df	95% CI
Initial	6.615	0.121	284.680	6.377–6.853
1 month	7.854	0.155	284.578	7.549–8.160
7 month	8.120	0.164	277.701	7.798–8.443
13 month	7.987	0.152	273.094	7.689–8.286

COPAQ scores show a significant increase from baseline to 7 months, with a slight decrease at 13 months, although they remain higher than the initial scores.

#### 3.3.4 Correlation matrix for covariance parameter estimates

The correlations between repeated measures at different time points were evaluated. The moderate to high positive correlations indicate consistency in scores over time (Table 5).

**Table 5:** Correlation matrix for covariance parameter estimates.

Parameter	UN (1.1)	UN (2.1)	UN (2.2)	UN (3.1)	UN (3.2)	UN (3.3)	UN (4.1)	UN (4.2)	UN (4.3)	UN (4.4)
UN (1.1)	1	0.557	0.164	0.522	0.157	0.137	0.485	0.144	0.124	0.105
UN (2.1)		1	0.577	0.921	0.562	0.501	0.864	0.537	0.478	0.431
UN (2.2)			1	0.537	0.952	0.830	0.506	0.913	0.796	0.720
UN (3.1)				1	0.578	0.566	0.947	0.557	0.545	0.495
UN (3.2)					1	0.953	0.548	0.966	0.919	0.836
UN (3.3)						1	0.539	0.925	0.969	0.886
UN (4.1)							1	0.566	0.553	0.533
UN (4.2)								1	0.950	0.916
UN (4.3)									1	0.969
UN (4.4)										1

## 4 Discussion

The main objective of this study was to evaluate whether the conceptualization of pain amongst young people aged 11–13 varies after participating in the specific Pain Science Education programme “Learning Pain” compared to a control group. This is the first Spanish trial to examine the pain conceptualization accuracy of children who receive a Pain Science Education programme versus a group control condition and the first study to investigate whether this change is sustained 1 year after programme delivery.

This study recorded the presence of pain based on participants’ responses to whether they had experienced pain in the past month. A total of 82% reported pain during this period, indicating that most participants either had pain at the time of the survey or recalled experiencing it recently and found it significant enough to mention. Given the adaptive role of pain in survival (58), it is unsurprising that such a high percentage reported it. The 18% who denied pain might have done so due to how they interpreted the experience rather than its absence. Pain may have been too minor or insignificant for them to report or even remember.

The results suggest that the “Learning Pain” programme significantly provide more accurate conceptualization of pain to young people compared to a control group. These results were maintained at 7 and 13 months, with the best results observed at 7 months.

This more accurate conceptualization of pain found at the 1-month assessment is consistent with that found in other studies that implemented Pain Science Education programme for students in the school setting to modify the concept of pain (22, 34, 39–41, 59). These immediate results in improving pain conceptualization are similar to those found by Mankelow et al. (40), in which pain knowledge was assessed using The Concept of Pain Inventory (COPI), although slightly lower than the results described by other authors (34, 41, 59). It is worth noting that the assessment tools used differ amongst these studies.

Most importantly, our data showed that at the 7-month assessment, the experimental group improved their pain conceptualisation results compared to the 1-month assessment. Indeed, all Pain Science Education interventions improve pain knowledge, but the few studies that analyse whether this knowledge persists over time typically find a decrease in knowledge unless reminders are provided. Louw et al. (39) assessed participants 6 months after the intervention and found better results in the group that received the intervention after two months. Martí et al. (41) analysed the degree of pain-related knowledge amongst adolescents after watching a brief educational video and found a deterioration in knowledge improvement between the immediate assessment and the 1-month assessment, indicating that “additional strategies are needed to help maintain the gained knowledge long-term.”

It should be noted that the present study is, to date, the only one that has assessed the long-term effects of a Pain Science Education programme intervention in a school setting. In the long-term assessment, the experimental group showed sustained improvement in conceptualisation of pain on the COPAQ which supports that the results indicate deep learning that is maintained at 13 months and could suggest that the results derived from the intervention measure conceptualisation change and not merely aspects related to the memorisation of new concepts.

It is worth mentioning that the “Learning Pain” programme was designed using a methodology that has been shown to help promote conceptual change (60). Thus, methodologies designed for this purpose, such as those proposed by Mankelow et al. (40) or the one in the present study, could be more suitable to facilitate pain concept change than those that only aim to memorise concepts. Since it was based on the students’ personal experiences with respect to pain, it was carried out with their active participation and used the memory, as it was done in two sessions separated by 1 month. It is well known that active retrieval promotes meaningful learning by connecting new information with existing schemas, making the learning more relevant and useful for the student (60).

The fact that the pain education programme was designed with consideration of the relationship between what was learned, and the students’ individual experiences might explain why the items that were more accurately answered by the experimental group compared to the control group at 7 and 13 months were related to more experiential issues, which might require more time to integrate. As Pate et al. (61) described, children construct their concept of pain through both knowledge about pain and with their individual experiences.

The control group showed a consistent 3% improvement in the COPAQ questionnaire at both 1-month and 7-month assessments compared to the initial evaluation. These findings suggest that the control group provided a stable and reliable basis for comparison with the experimental group and for discussing results derived from the intervention. These results differ from Louw et al. (39), where the control group without Pain Science Education showed an 11% improvement in the NPQ questionnaire, compared to 14% in the experimental group—a 3% difference in pain knowledge. In contrast, the present study found a 15% difference in COPAQ scores between experimental and control groups.

To understand the differences in results between the different studies, it may be important to consider the assessment tool used and the content developed in the educational programmes. The use of questionnaires not validated for children and not specifically designed to assess the concept of pain could make it difficult to compare the results obtained in studies that implement Pain Science Education.

Louw et al. used the Pain Neurophysiology Questionnaire (NPQ) with language adaptations for easier understanding, but it is not validated for children. The 2018 study added 13 NPQ questions, whilst the 2020 study included 12 (34, 39). Similarly, Wager et al. (22) and Martí et al. (41) used unvalidated tools created specifically for their studies. In contrast, the present study and Mankelou et al. (40) used validated questionnaires—COPI and COPAQ—to measure pain conceptualization in children and adolescents (43, 62). This validation may explain the greater similarity in their results compared to other studies.

Both questionnaires were designed to assess the conceptualization of pain in children and adolescents (43, 62). In contrast, the NPQ was not developed as a tool for use with children and focuses more on pain knowledge than on pain conceptualization (63). Given that Pain Science Education is based on the theory of conceptual change (16), it might be pertinent to seek greater consensus and rigour in the use of assessment tools to measure the effectiveness of Pain Science Education programmes in research studies.

This study based the contents of the “Learning Pain” programme on the objectives considered essential for paediatric Pain Science Education programmes (57). In other Pain Science Education programmes, additional contents were included, such as peripheral sensitization, central sensitization, physiological stress response, and endocrine responses in pain (34, 39, 59). Although these topics are well documented in Pain Science Education programmes for adults (12, 13) and are assessed to the NPQ assessment tool, they may differ from those advised to promote a change in the conceptualization of pain in children and adolescents (50, 57, 62).

The results of this study show that the implementation of the Learning Pain programme increased pain conceptualization in children and adolescents aged 11–13 years, achieving an understanding more aligned with the current pain paradigm. However, the effect that this increase in conceptualization may have on maladaptive behaviours or on the pain itself is unknown and thus further work is needed to explore the potential impact of bringing Pain Science Education into the curriculum is needed.

### 4.1 Limitations and future lines of research

This study was conducted in a very specific age group (11–13 years) as it was carried out in the first year of middle school. Therefore, the results cannot be generalised to other ages. Future research could analyse the effectiveness of the programme in other age groups. This could be beneficial in terms of establishing Pain Science Education programmes throughout the school years within the framework of school health campaigns.

In this study, the analysis of conceptualization according to the persistence of pain was not carried out, which is a limitation in terms of understanding how this change in conceptualization affects the participants’ experience of pain. Future lines should investigate whether possible changes in participants’ pain, as well as what is the minimum clinically significant difference for the COPAQ questionnaire.

Furthermore, this study has not analysed the possible improvement of maladaptive behaviours in participants with pain or how future maladaptive behaviours in pain could be prevented. It would be interesting if future studies could explore which maladaptive behaviours young people have and how these could be modified with this type of intervention.

A possible explanation for the decrease in the experimental group’s results at 13 months compared to the results at 7 months could be the lack of an educational intervention for families during the study. Considering the importance of family influence on children’s pain (64), the participation of parents in Pain Science Education programmes framed within Public Health prevention campaigns could be fundamental for achieving greater effectiveness. It appears essential to involve parents in paediatric pain education to optimise pain-related outcomes for children. Additionally, it may be beneficial to explore whether some parents need extra pain education that addresses their own beliefs, perceptions, and coping strategies as part of managing their child’s pain (24).

## 5 Conclusion

Students who received the “Learning Pain” programme (experimental group) obtained better scores on the COPAQ questionnaire increase from baseline to 7 months, with a slight decrease at 13 months, although they are still higher than at baseline, resulting in a more accurate conceptualization of pain compared to the control group, which maintained a stable mean score during the assessments. Therefore, the specific Pain Science Education programme “Learning Pain” was associated with improved conceptualization of pain in adolescents aged 11–13 years in the short, medium, and long term.

## Data Availability

The raw data supporting the conclusions of this article will be made available by the authors, without undue reservation.Unpublished protocol will be made available upon reasonable request from Laura Menés Fernández (https://orcid.org/0000-0001-8599-4478). Reuse without ethical approval is not permitted. A data-sharing agreement will require a commitment to using the data only for specified research purposes, to securing the data appropriately and to destroying the data after a nominated period.
