# Supplementary figures and images for: Effectiveness of a pain science education programme in middle school students: a randomised controlled trial
Source: Front Public Health. 2025 Jan 22;12:1423716. doi: 10.3389/fpubh.2024.1423716 (PMC11794317; doi:10.3389/fpubh.2024.1423716)

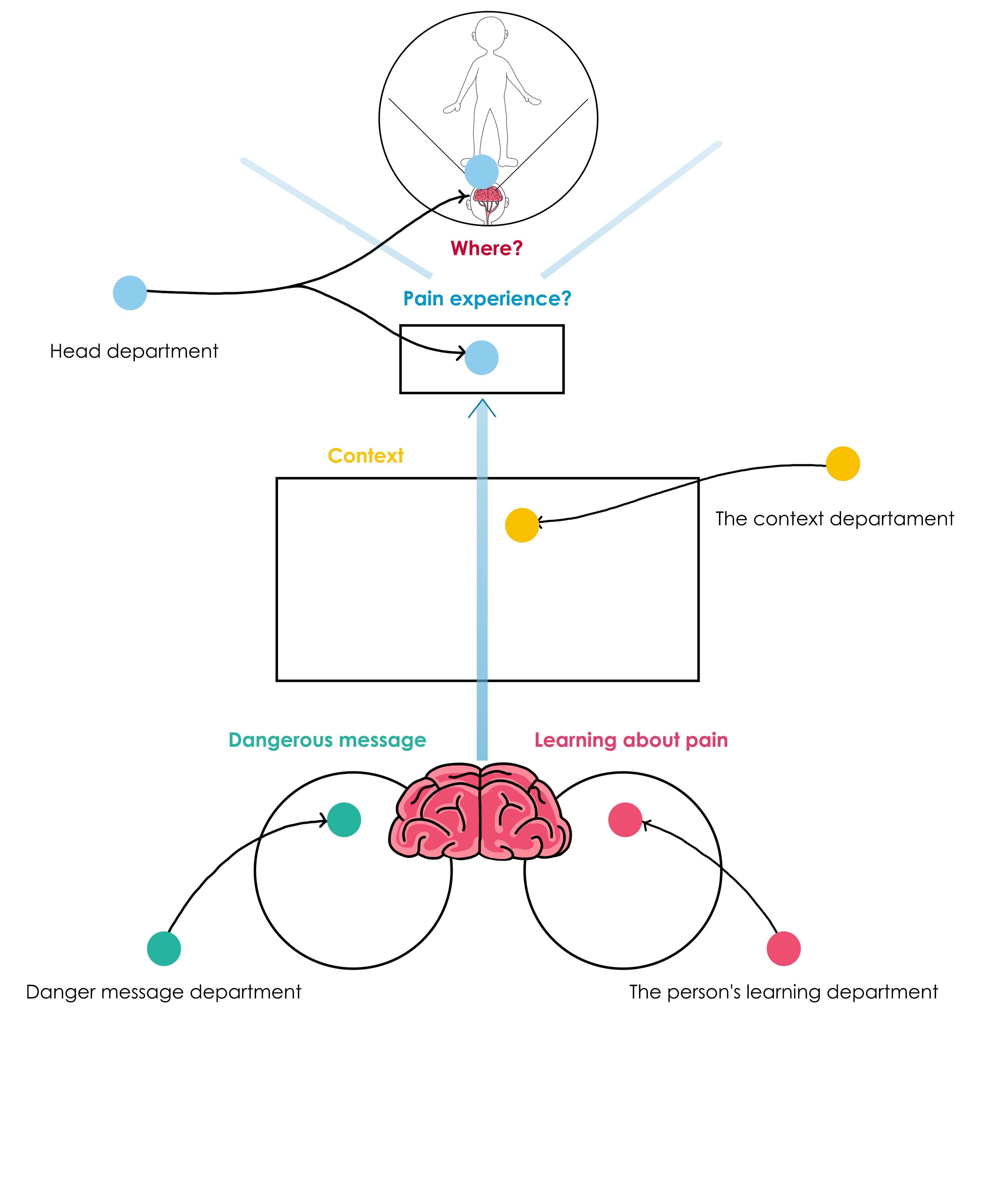

Supplement: Supplementary file 1 [file Image_1.JPEG]

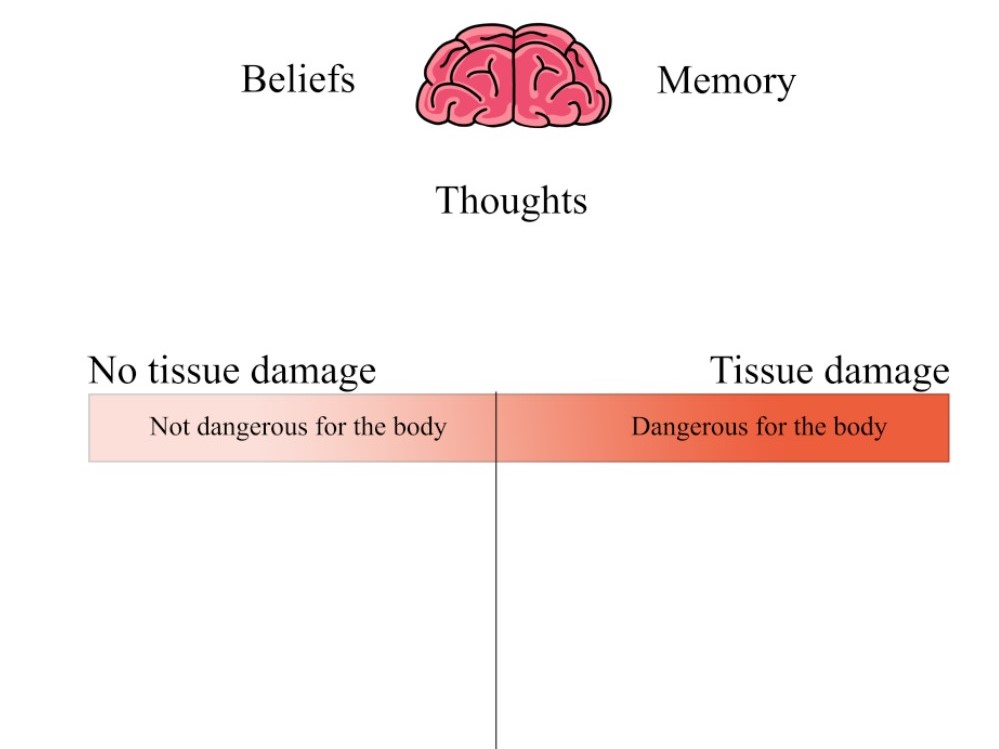

Supplement: Supplementary file 2 [file Image_2.JPEG]

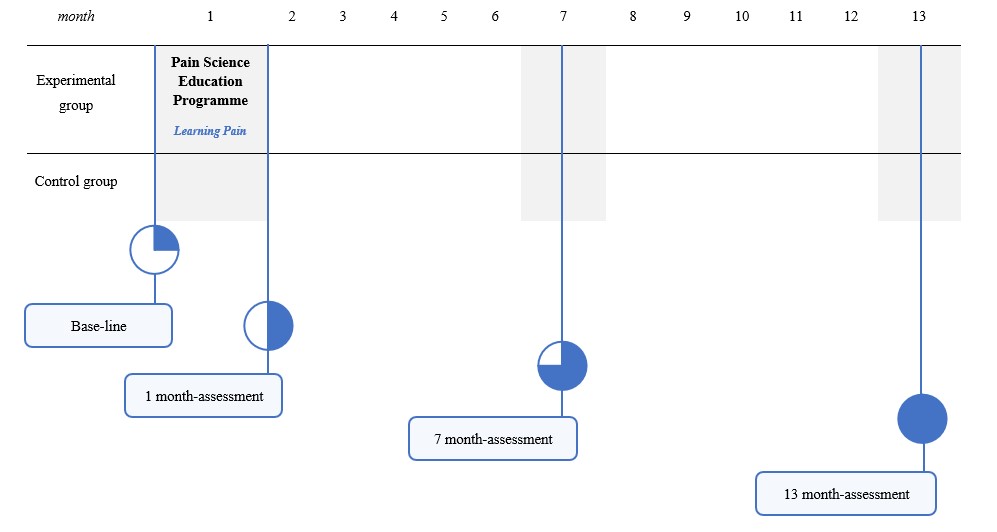

Supplement: Supplementary file 3 [file Image_3.JPEG]

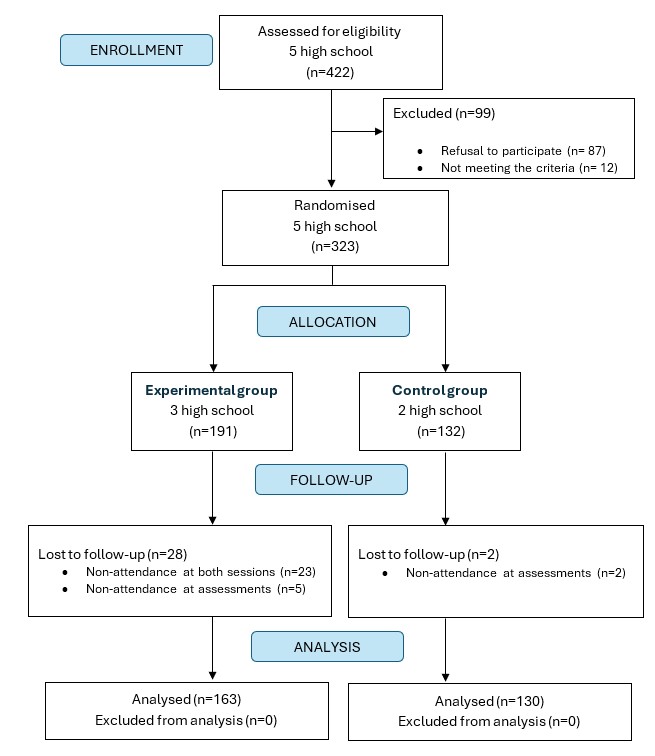

Supplement: Supplementary file 4 [file Image_4.JPEG]
